# Editorial: Visual Snow: Old Problem, New Understanding

**DOI:** 10.3389/fneur.2022.884752

**Published:** 2022-04-15

**Authors:** Owen B. White, Joanne Fielding, Victoria Susan Pelak, Christoph J. Schankin

**Affiliations:** ^1^Central Clinical School, Faculty of Medicine, Nursing and Health Sciences, Monash University, Melbourne, VIC, Australia; ^2^School of Medicine, University of Colorado, Aurora, CO, United States; ^3^Department of Neurology, Inselspital, Bern University Hospital, University of Bern, Bern, Switzerland

**Keywords:** Visual snow syndrome, sensory perception, sensorimotor interaction, functional image analysis, central processing

Visual snow syndrome (VSS) is a well known disturbance of visual perception. It has been misdiagnosed over the years as either being an untreatable form of migraine or, conversely, a manifestation of psychological disturbance. It was eventually recognized as a separate entity in 1995 (1) and was subsequently codified as a syndrome in a series of papers from 2012 to 2014 (2–5), and expanded on as part of a more widespread disturbance of sensory processing on the basis of its association with numerous other sensory disturbances (6, 7). It is described as a very rare entity (8) and yet recent studies have suggested that it may affect as much as 2.2% of the community (9).

VSS is a clinical diagnosis with the main criterion of persistent pan-field “TV-snow” or “pixilation,” extending beyond 3 months, in conjunction with two of either photophobia, enhanced entoptic phenomena (e.g., blue field phenomena, floaters), palinopsia, or nyctalopia (4, 5). Numerous other non-visual or even non-perceptual symptoms have been identified, including migraine, tinnitus, vestibular disturbance (consistent with persistent perceptual postural disorder), fibromyalgia, migratory paresthesia, and endogenous perceptual phenomena such as depersonalization and derealization (6, 7, 10).

Recent work has demonstrated both structural (11–15) and physiological/functional signatures of the disorder (16–18) (Foletta et al.; Solly et al.), confirming this as a separate disorder from migraine and, in conjunction with the above quoted studies, introduces the concept of impaired central processing as a new group of disorders deserving of study, as well as providing a means for study.

Thus, the recent interest in visual snow has enfranchised a group of patients previously mislabeled as neurotic or having untreatable migraine. It has also provided a means for evaluating potential treatment strategies, permitting trials going forward.

This Research Topic of Frontiers in Neurology, Section Neuro-Ophthalmology is focused on the current advances in the field of visual snow research. It demonstrates the progress made in clinical, paraclinical, and experimental aspects with the ultimate aim to reduce symptoms and improve quality of life in these patients.

Currently, it is still difficult to determine what drives patients to develop VSS later in life or early during childhood. In early studies, eye examinations were in general normal (1, 5) but it has remained unclear how much ophthalmic examination is necessary. The retrospective study from this Research Topic reviewed detailed ophthalmology exams in 52 patients and revealed that patients presenting with typical VSS may not need more than the standard neuro-ophthalmologic examination and automated perimetry (Vaphiades et al.). From the beginning, it has been suggested that VSS might be a migrainous phenomenon, essentially driven by the comorbidity and the more severe presentation in patients who have both (5, 19). The pathophysiological link has been investigated by Eren et al. who, in an electrophysiological study, assessed magnetic suppression of perceptual accuracy, which is reduced in patients with migraine with aura and chronic migraine (19). In patients with VSS, this is not the case, suggesting that occipital cortex inhibition is not affected, and that the mechanisms of VSS and migraine with aura are different. In contrast, a predisposition to VSS might exist for patients with episodic visual snow as nicely shown in the case report by Puledda et al. (20). In this manuscript, a 44-year-old man had an acute stroke in the right superior cerebellar artery territory and immediately developed all the symptoms of VSS. Prior to the stroke, he had the same symptoms in an episodic manner about once per month. This means that episodic visual snow might not be so uncommon (21) and may be a risk factor for VSS. This is important for counseling patients with episodic visual snow.

Another focus of this Research Topic was brain physiology in VSS. Harris et al. analyzed double-pulse visual evoked potentials (VEPs) and found a differential pattern of VEP attenuation and potentiation in one patient suggesting that multiple mechanisms of neuronal responsiveness to visual stimulation might exist in VSS. In a psychophysical experiment, attention was investigated by Foletta et al., who assessed eye movements toward and away from target stimuli using an “inhibition of return” paradigm. This study raises the potential for a distinct saccadic behavioral profile in VSS that might serve as a biomarker for VSS for future therapeutic studies, and suggests that attention is impacted in VSS.

From a neuroimaging perspective, Michels et al. investigated the white matter in VSS. They found widespread alterations in prefrontal, temporal, and occipital areas, supporting the fact that atypical visual processing and conceptualization might be an important mechanism in this disorder. Taken together, VSS might be regarded as a particular form of network disorder as carved out in a systematic review by Klein and Schankin (7). The authors presented and combined what is known from clinical, neurophysiological, functional, and structural imaging in visual and extra-visual areas. They conclude that VSS is a network disorder with key structures in pre-cortical and attentional networks, where filtering and prioritizing might happen.

Despite all these advances in our clinical and pathophysiological understanding of VSS, therapy remains a substantial challenge. Against this background, Solly et al. profiled patients with VSS in respect of psychiatric and neuropsychological symptoms with a focus on depression, anxiety, depersonalization, sleep, fatigue, and quality of life. Although VSS is clearly not a psychiatric disorder, patients with VSS show high rates of psychiatric symptoms and a reduction of quality of life (Solly et al.). For clinical practice, this is highly important. Currently, we cannot offer evidence-based treatment for the key visual symptoms of VSS. However, the psychiatric comorbidities can be identified and addressed specifically with the aim of increasing overall quality of life in our patients. Another important therapeutic option is non-pharmacological. Hepschke et al. (22) applied intuitive colorimetry in a psychophysical experiment. They found that VSS discomfort exacerbates with short-wave (i.e., “blue”) cone activation, which is important for two reasons. First, the koniocellular pathway might be affected in VSS; and second, intuitive colorimetry starting in the blue filter area should be offered to patients with the aim of reducing visual discomfort. Although symptomatic therapy and consideration of psychiatric comorbidity is important, etiological treatment will be the ultimate goal in VSS research. Also in this Research Topic, Grande et al. published a promising protocol for an open-label pilot trial for a 10-day treatment protocol for VSS with transcranial magnetic stimulation. The authors report some of the burdens associated with TMS treatment protocols, including the need for multiple consecutive treatment days and the current COVID-19 pandemic. Outcome measures being used include psychophysical perceptual measures, previously investigated in VSS by McKendrick et al. (23), that include assessment of center-surround contrast suppression and luminance increment thresholds in noise. Results of the TMS protocol are expected in the next year.

In the last decade, the establishment of clinical criteria for VSS has taken the disorder out of the area of psychiatric or psychological disease, as shown in this Research Topic, and set the stage for a rapid growth in research in many domains of neurology and neurosciences that might 1 day lead to a better understanding of and treatment for VSS and other disorders with similar underlying “network” mechanisms. Our previous nodal concept of cerebral function is inadequate to explain the brain's function. We need to move away from the Charcot-based evaluation of cerebral function, recognizing that it represents no more than 10% of cerebral circuits, and look at more in-depth studies. We must reorientate toward a concept of interactive networks which are neither sensory nor motor but are in fact sensorimotor. There are afferent components, efferent components, and central processing, about which we have much to learn but for which we have sufficient tools to make a start. Visual snow syndrome is a condition where we can start to gain these insights, and it will be exciting to go along with the development of this field in the coming years.

## Author Contributions

OW was responsible for setting up the introduction to the editorial and subsequent combination of all author contributions. JF and VP reviewed articles for the Research Topics and reviewed the manuscript. CS reviewed articles for the Research Topics, reviewed the manuscript, and substantially modified the initial concept of the editorial. All authors contributed to the article and approved the submitted version.

## Conflict of Interest

The authors declare that the research was conducted in the absence of any commercial or financial relationships that could be construed as a potential conflict of interest.

## Publisher's Note

All claims expressed in this article are solely those of the authors and do not necessarily represent those of their affiliated organizations, or those of the publisher, the editors and the reviewers. Any product that may be evaluated in this article, or claim that may be made by its manufacturer, is not guaranteed or endorsed by the publisher.
