# Corrigendum: Editorial: Visual Snow: Old Problem, New Understanding

**DOI:** 10.3389/fneur.2022.969405

**Published:** 2022-07-01

**Authors:** Owen B. White, Joanne Fielding, Victoria Susan Pelak, Christoph J. Schankin

**Affiliations:** ^1^Central Clinical School, Faculty of Medicine, Nursing and Health Sciences, Monash University, Melbourne, VIC, Australia; ^2^School of Medicine, University of Colorado, Aurora, CO, United States; ^3^Department of Neurology, Inselspital, Bern University Hospital, University of Bern, Bern, Switzerland

**Keywords:** Visual snow syndrome, sensory perception, sensorimotor interaction, functional image analysis, central processing

In the published article, the reference for Reference 20 was incorrectly written as “Puledda F, Schankin C, Goadsby PJ. Visual snow syndrome: a clinical and phenotypical description of 1,100 cases. *Neurology*. (2020) 94:e564–74. doi: 10.1212/WNL.0000000000008909”. It should be “Puledda F, Villar-Martínez MD, Goadsby PJ. Case report: transformation of visual snow syndrome from episodic to chronic associated with acute cerebellar infarct. *Front Neurol*. (2022) 13:811490. doi: 10.3389/fneur.2022.811490”.

The authors apologize for this error and state that this does not change the scientific conclusions of the article in any way. The original article has been updated.

## References

Puledda F, Villar-Martínez MD, Goadsby PJ. Case report: transformation of visual snow syndrome from episodic to chronic associated with acute cerebellar infarct. *Front Neurol*. (2022) 13:811490. doi: 10.3389/fneur.2022.811490

## Publisher's Note

All claims expressed in this article are solely those of the authors and do not necessarily represent those of their affiliated organizations, or those of the publisher, the editors and the reviewers. Any product that may be evaluated in this article, or claim that may be made by its manufacturer, is not guaranteed or endorsed by the publisher.

